# Retraction: Photocatalytic membranes prepared by embedding porous Zr-doped SiO_2_ shell/TiO_2_ core particles with expanded channels into polyvinylidene fluoride for cleaning wastewater

**DOI:** 10.1039/d2ra90013k

**Published:** 2022-02-16

**Authors:** Laura Fisher

**Affiliations:** Royal Society of Chemistry Thomas Graham House, Science Park, Milton Road Cambridge UK

## Abstract

Retraction for ‘Photocatalytic membranes prepared by embedding porous Zr-doped SiO_2_ shell/TiO_2_ core particles with expanded channels into polyvinylidene fluoride for cleaning wastewater’ by Yuqing Zhang and Pinjing Li, *RSC Adv.*, 2015, **5**, 98118–98129, DOI: 10.1039/C5RA16319F.

The Royal Society of Chemistry hereby wholly retracts this *RSC Advances* article due to concerns with the reliability of the data in the published article.

The TEM in Fig. 3b, which represents EC-ZSTs, is a duplicated, translated and scaled version of the TEM in Fig. 3e, which represents ZSTs. The TEM in Fig. 3d, which represents ZSTs, was previously published by the authors in a *Separation and Purification Technology* article to represent Zr-SVTs.^1^ The TEM in Fig. 3f, which represents ZSTs, was previously published by the authors in a *Separation and Purification Technology* article to represent Zr-SVTs and in a *Journal of Colloid and Interface Science* article to represent Zr-SVT-4.^1,2^ The methods sections of this *RSC Advances* article and the *Separation and Purification Technology* and *Journal of Colloid and Interface Science* articles describe materials synthesised using different protocols.^1,2^

The pore size distribution inset in Fig. 5 for ZSTs was previously published by the authors in a *Journal of Colloid and Interface Science* article to represent Zr-SVT-4 with a different corresponding nitrogen adsorption–desorption isotherm.^2^ In addition, the data points at low and high partial pressures in the isotherm in Fig. 5 in this *RSC Advances* article appear to be identical to the data points at low and high partial pressures in the isotherm in the *Journal of Colloid and Interface Science* article.^2^ The nitrogen adsorption–desorption isotherm in Fig. 5 for ZSTs in this *RSC Advances* article was also previously published by the authors in a *Chemical Engineering Journal* article for ZVTs.^3^

The EDX spectrum in Fig. 7 for EC-ZSTs was previously published by the authors in a *Separation and Purification Technology* article for Zr-SVTs,^1^ in a *Journal of Colloid and Interface Science* article for Zr-SVT-4,^2^ and in a *Chemical Engineering Journal* article for ZVTs,^3^ and was later published by the authors in a *Chemical Engineering Journal* article for SYFZr-Tis.^4^ The methods sections of this *RSC Advances* article and the *Separation and Purification Technology*, *Journal of Colloid and Interface Science* and *Chemical Engineering Journal* articles describe materials synthesised using different protocols.^1–4^

The FT-IR spectra in Fig. 8 for EC-ZSTs and STs was previously published by the authors in a *Journal of Colloid and Interface Science* article for Zr-SVT-4 and SVTs.^2^

The XRD pattern for TiO_2_ in Fig. 9a has two sets of repeating fragments along the baseline. In addition, the XRD patterns in Fig. 9a and b for TiO_2_ and EC-ZSTs were also previously published by the authors in a *Separation and Purification Technology* article for TiO_2_ and Zr-SVTs. The methods sections of this *RSC Advances* article and the *Separation and Purification Technology* article describe materials synthesised using different protocols for EC-ZSTs and Zr-SVTs.^1^

The elemental composition data in Table 1 for EC-ZSTs is the same as reported for Zr-SVTs in a *Separation and Purification Technology* article and for Zr-SVT-4 in a *Journal of Colloid and Interface Science* article by these authors.^1,2^ The methods sections of this *RSC Advances* article and the *Separation and Purification Technology* and *Journal of Colloid and Interface Science* articles describe materials synthesised using different protocols.^1,2^

In addition, there are significant portions of unattributed text overlap throughout this *RSC Advances* article with other published articles, primarily a *Materials & Design* article^6^ and a *Chemical Engineering Science* article^5^ by these authors.

The authors were contacted for comment, but their response was not sufficient to explain the concerns with this article. Raw data was requested, but the authors did not provide it.

Given the significance of the concerns about the validity of the data and the lack of raw data, the findings presented in this paper are no longer reliable.

Yuqing Zhang and Pinjing Li oppose this retraction.

Signed: Laura Fisher, Executive Editor, *RSC Advances*

Date: 11^th^ February 2022

## Supplementary Material
